# Supplementary material for: Infection patterns in simple and complex contagion processes on networks
Source: PLoS Comput Biol. 2024 Jun 10;20(6):e1012206. doi: 10.1371/journal.pcbi.1012206 (PMC11192313; doi:10.1371/journal.pcbi.1012206)
Supplement: S1 Text — Supporting information is provided in a separate pdf file. It contains additional analyses on infection pattern, attack rate, spreader and receiver index executed for the primary school dataset used in the main text and for additional datasets. (PDF) [file pcbi.1012206.s001.pdf]

Supplementary material for
Infection patterns in simple and complex contagion processes on networks

Diego Andrés Contreras, Giulia Cencetti, Alain Barrat

A. INFECTION PATTERN ASYMMETRY AND SIMILARITY WITH ADJACENCY MATRIX

We report in Fig. S1 the similarity of the infection patterns with the weighted adjacency matrix (left panels) for the simple, simplicial, and threshold models, as a function of the model parameters. The similarity is high but less than 1, and lower for the simplicial and especially the threshold model. We also observe that similarity slightly decreases when increasing R_0 for the model of simple spread and when decreasing θ in threshold model, i.e. when increasing the infection probability on each link. In extreme cases, indeed, the infection probabilities saturate and contagions are possible on each existing link independently on its weight. Smoothing out the differences between weights implies reducing the similarity of the infection patterns with the adjacency matrix. In the simplicial model instead the similarity with the adjacency matrix decreases when increasing $\beta_\Delta/\beta_|$, i.e. when infection events are favored on triangular interactions with respect to link interactions.

The right panels of Fig. S1 report the distributions of the symmetry of the contact patterns, as quantified by the ratio between link weights in opposite directions of each pair of connected nodes, for the three models of contagion. Specifically, for each edge (i, j) we report the infection probability in the direction that has been less used divided by the infection probability in the opposite direction, $\min(C_{ij}, C_{ji})/\max(C_{ij}, C_{ji})$. For symmetric infection patterns, this quantity is 1 for each edge, and 0 in the extreme case of an edge in which the contagion is observed only in one of the two possible directions.

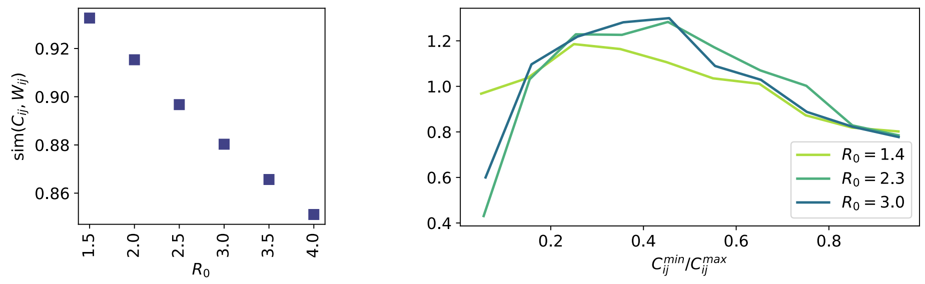

(a)Simple

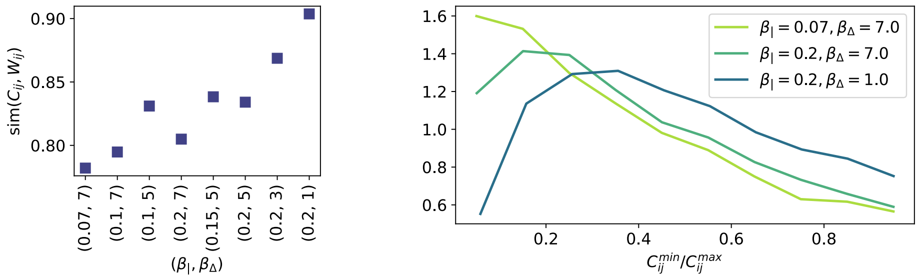

(b)Simplicial

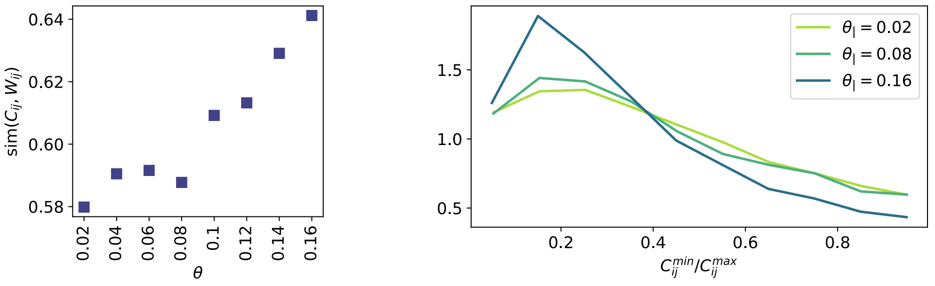

(c)Threshold

FIG. S1. Cosine similarity between infection pattern **C** and weighted adjacency matrix **W** on the left, and distribution of symmetry in infection pattern on the right.

B. EPIDEMIC CURVES IN SIMPLE CONTAGION

Figure S2 shows the temporal evolution of the number of infected in various models of simple contagion. These involve only infected for the SIR model, and exposed and infected for the other models. The time evolution of the epidemic is clearly different across models.

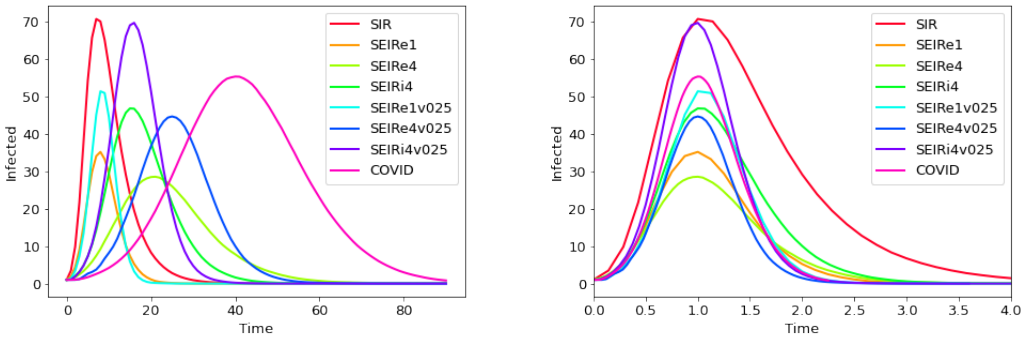

(a)

(b)

FIG. S2. Number of infected individuals vs. time for different models of simple contagion (I for SIR model, E+I for all the SEIR models), with $R_0 = 3.0$. In the right panel the time has been normalized so as to have the maximum peak at $t = 1$ for all models.

C. WEIGHTED ADJACENCY IN NETWORKS AND IN SIMPLICIAL COMPLEXES

In the main text we mentioned that for real networks higher-order and pairwise interactions largely overlap, i.e., nodes connected in groups with large weights are typically also connected by links with large weights. To show this we report in Fig. S3 a scatterplot where for each pair of nodes (i,j) we show the pairwise weight W_{ij} and the second-order weight of all the triads that the pair is involved in, $\sum_k W^{\Delta}_{ijk}$, for the five different data sets. The Pearson correlation is reported in each panel.

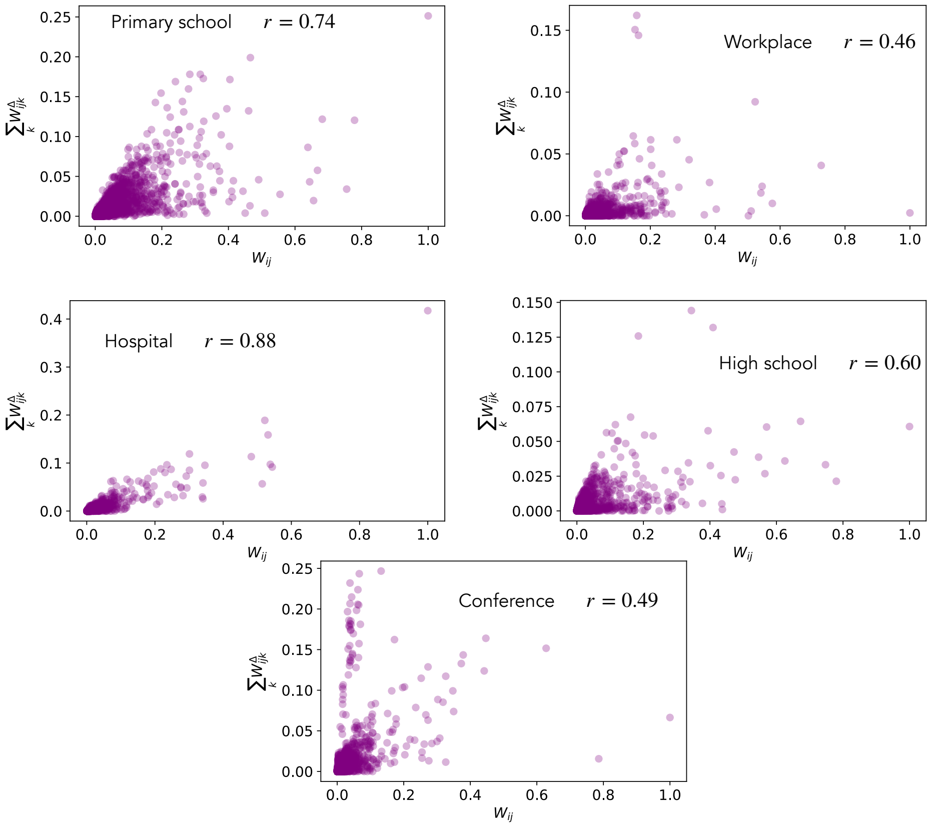

FIG. S3. Correlation between link weight and triads weights for each couple of nodes in each network.

D. UNCORRELATED HYPERGRAPHS

As mentioned in the main text and illustrated in the previous section, the hypergraphs built from real data sets present a high correlation between the weights of first and second order interactions: if a hyperedge (i, j, k) has a high (low) weight we typically observe that the pairwise interactions (i, j), (j, k) and (k, i) are also associated to high (low) weights. This can explain the high values of similarity that we observed in section II C between infection patterns obtained at varying parameters (β, β_Δ). Indeed, changing such parameters leads to a shift from having more contagion events through links than in triadic interactions (for high β, low β_Δ) to the reverse situation (at low β and high β_Δ). However, the fact that the weights of links and triadic interactions are correlated means that this shift does not lead to strongly different infection patterns.

To better show this point, we perform here an additional set of numerical experiments. We first build synthetic hypergraphs in which we remove the correlations between the weights of the links and of the triads. To this aim, we consider the original hypergraph, keep its structure fixed (list of links and triads), keep as well the weights of the triads fixed, but reshuffle the weights of the links. Note that we still have a correlation between the first and second order interactions as those largely overlap in the data. However, one can have a triad with a large weight composed of links with small weights and vice-versa, with overall no weight correlations. This absence of correlations is checked in Fig. S4(f) which, analogously to Fig. S3, is a scatterplot reporting for each link its weight and the sum of weights of all triads involving that link: we obtain a very small coefficient correlation (here for the primary school data set), while it was of 0.74 in the original data (see Fig. S3). We then simulated the simplicial contagion process on the hypergraph with no weight correlations, measured the infection patterns for the same parameter values as in the main text, and performed the same comparisons between the patterns obtained for different parameters.

Figure S4 reports the corresponding results for the primary school data set, in a way analogous to Fig. 5 of the main text. Note that, to obtain panel (e), we simulate the simple contagion process using the same link weights as in the reshuffled hypergraph. The similarity values we obtain are notably lower than in the original hypergraph, confirming that the high similarity observed in the real hypergraphs is largely due to the correlations between the weights of the first and second order interactions.

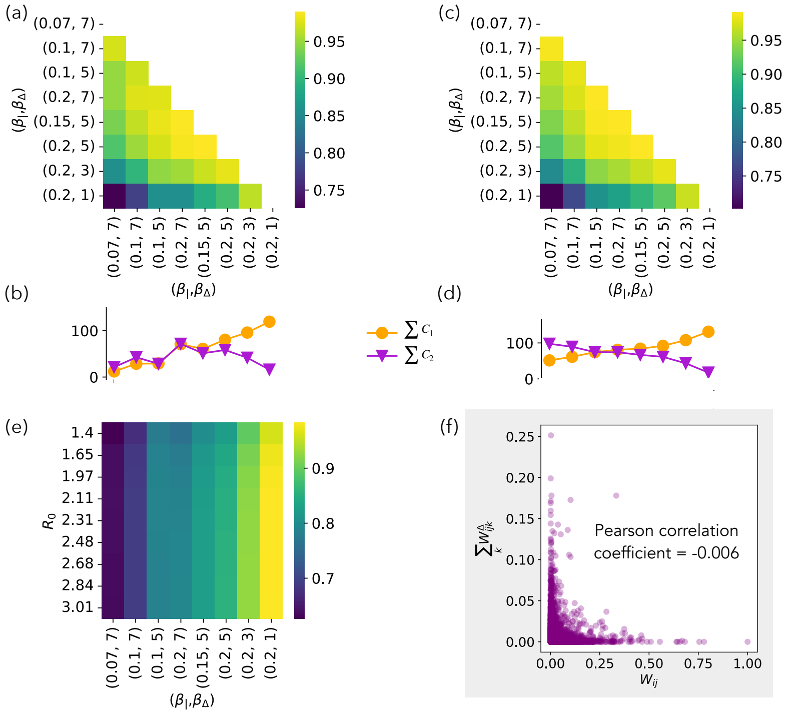

FIG. S4. Infection patterns for simplicial contagion models simulated on the hypergraph obtained from the primary school data set and with reshuffled weight links. Panels (a) to (e) are built similarly to the ones of Fig. 5 of the main text. (a): Cosine similarity between infection patterns obtained with different combinations of $\beta_|$ and β_Δ. (b): Number of contagions taking place via first and second order hyperedges in the simulations of the previous panel. (c): Same as (a) but when the infection patterns are computed using only simulations with attack rate between 0.6 and 0.7. (d): Number of contagions taking place via first and second order simplices in the simulations of the previous panel. (e): Cosine similarity between the infection patterns of the simplicial contagion model (for the same range of values of $\beta_|$ and β_Δ) and simple contagion (for several values of R_0). (f): Correlation between link weight and triads weights for each couple of nodes in the hypergraph with reshuffled link weights, similarly to Fig. S3.

E. DISTRIBUTIONS OF FINAL ATTACK RATES IN THE SIR SIMPLE CONTAGION MODEL

In simple contagion processes, the final attack rate (number of nodes that have been infected at the end of the process) depends on the stochastic realizations, even at fixed parameters. To each value of R_0, we can thus associate a distribution of final attack rate values, which we build by collecting the final attack rates of all the numerical simulations with the same R_0. Figure S5 shows the obtained distributions for the SIR model and different choices of R_0, built with 1000 stochastic runs for each R_0. In particular, panel (a) shows the attack rate for the values of R_0 used in Fig. 3(c) of the main text. On the right we report for each R_0 the percentage of these simulations that yield a final attack rate between 0.75 and 0.85. For instance, only about 3% of runs fall into this interval for $R_0 = 1.65$. Panel (b) analogously shows the attack rate for the lower values of R_0 used in Fig. 3(d-e) and, on the right, the percentage of simulations that fall into the intervals $(0, 0.2)$ and $(0.5, 0.6)$.

The infection patterns used in Fig. 3(c) are the result of the average over 1000 simulations for each R_0, which means that we actually perform a much larger number of simulations, in fact as many as needed until we have obtained 1000 simulations with final attack rate in the desired range. For instance, for $R_0 = 1.65$ we need around 33000 simulations. The infection patterns used in Fig. 3 (d) and (e) are instead averaged over 10000 and 50000 runs, respectively. So for instance with $R_0 = 1.72$, in order to find enough instances with attack rate between 0.5 and 0.6 we need around 200000 simulations.

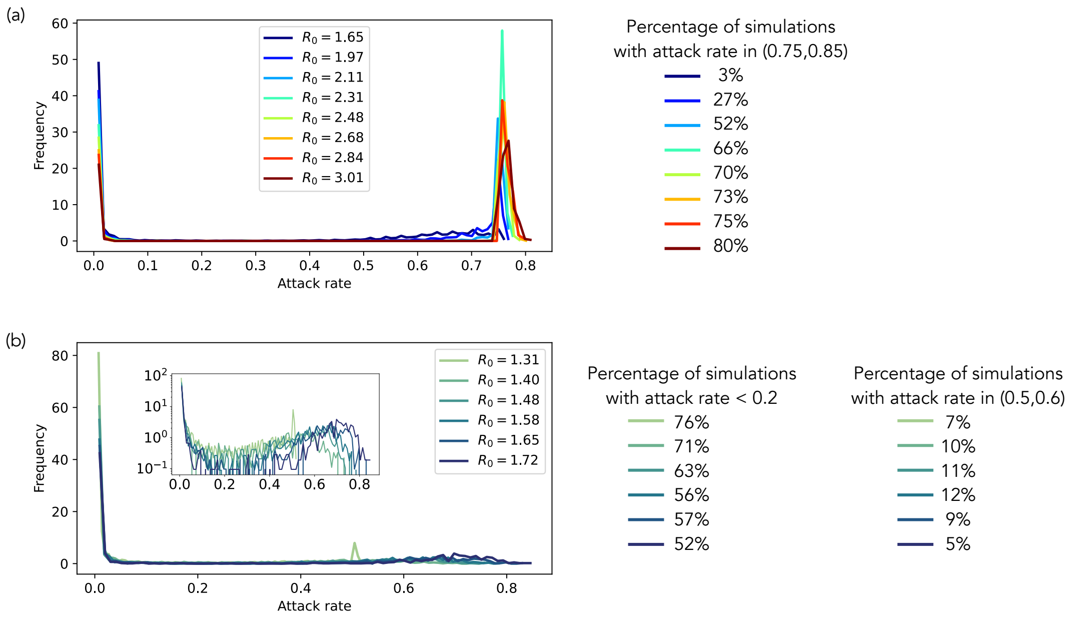

FIG. S5. Distributions of final attack rates in numerical simulations of SIR simple contagion processes for different values of R_0, for the primary school data set. Each distribution is obtained from 1000 numerical simulations with random initial conditions. In panel (b) the inset shows the same distributions with a logarithmic y scale.

F. SPREADER AND RECEIVER INDICES

Starting from the infection pattern matrix $\mathcal{C}$ we can define two indicators that characterize the role of each node by quantifying their ability to spread and to receive:

- **Spreader index** $s_i = \sum_j C_{ij}$: expected number of direct infections produced by node i.
- **Receiver index** $r_i = \sum_j C_{ji}$: expected number of direct infections received by node i.

We report the two indices for simple, simplicial, and threshold contagion in Figs. S7, S8, S6, S9, S10 and S11, showing the relation between s_i and r_i, and the similarity (cosine and ranking) of each of them at varying parameters. We notice that these measures, even if they are correlated among them, do not coincide. This is due to the asymmetry of matrix $\mathbf{C}$ and implies that having a large probability of being infected does not necessarily correspond to a large spreading ability, as it has been recently discussed in literature [1].

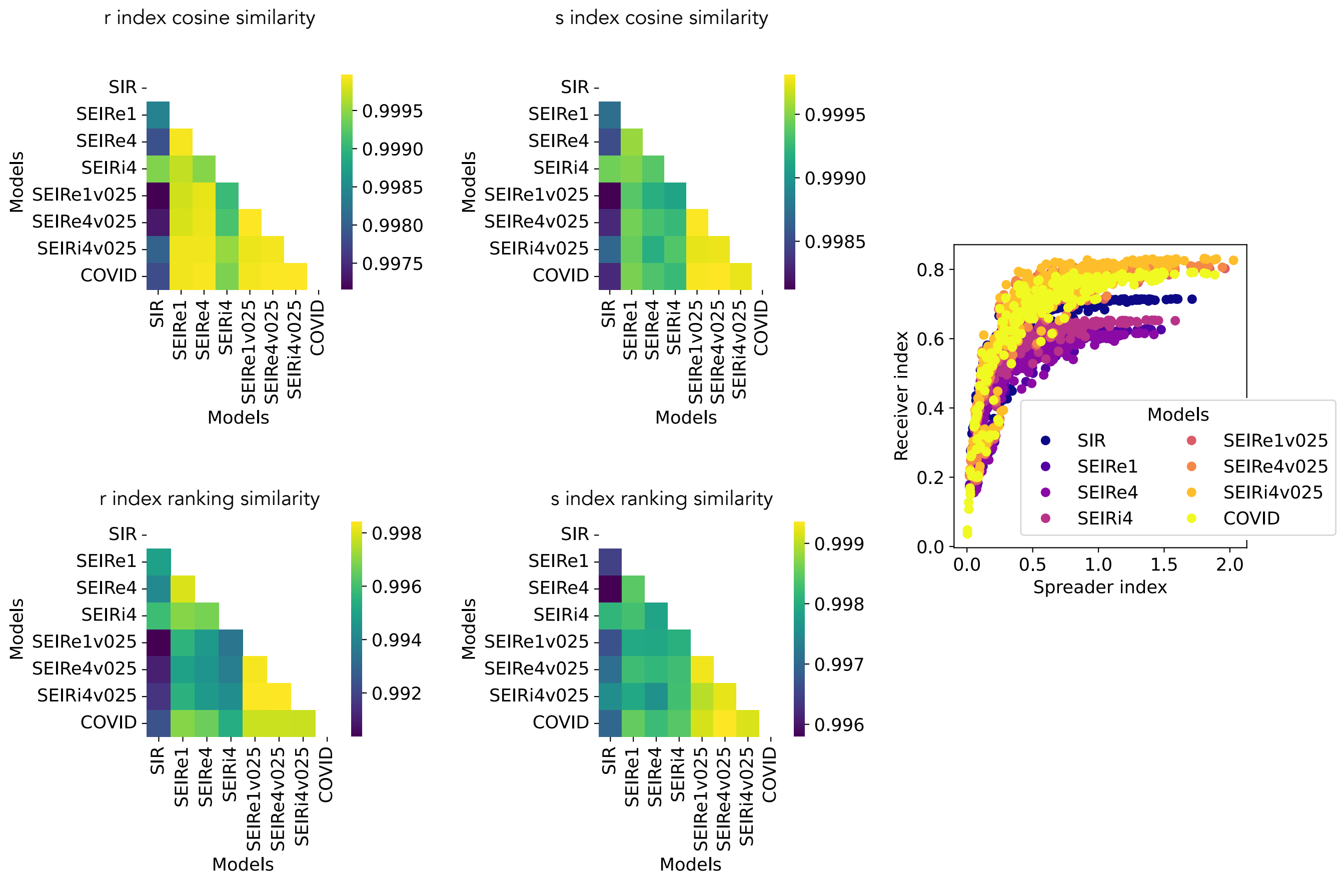

FIG. S6. Receiver and spreader indices across models of simple contagion with fixed $R_0 = 2.5$ for the primary school data set.

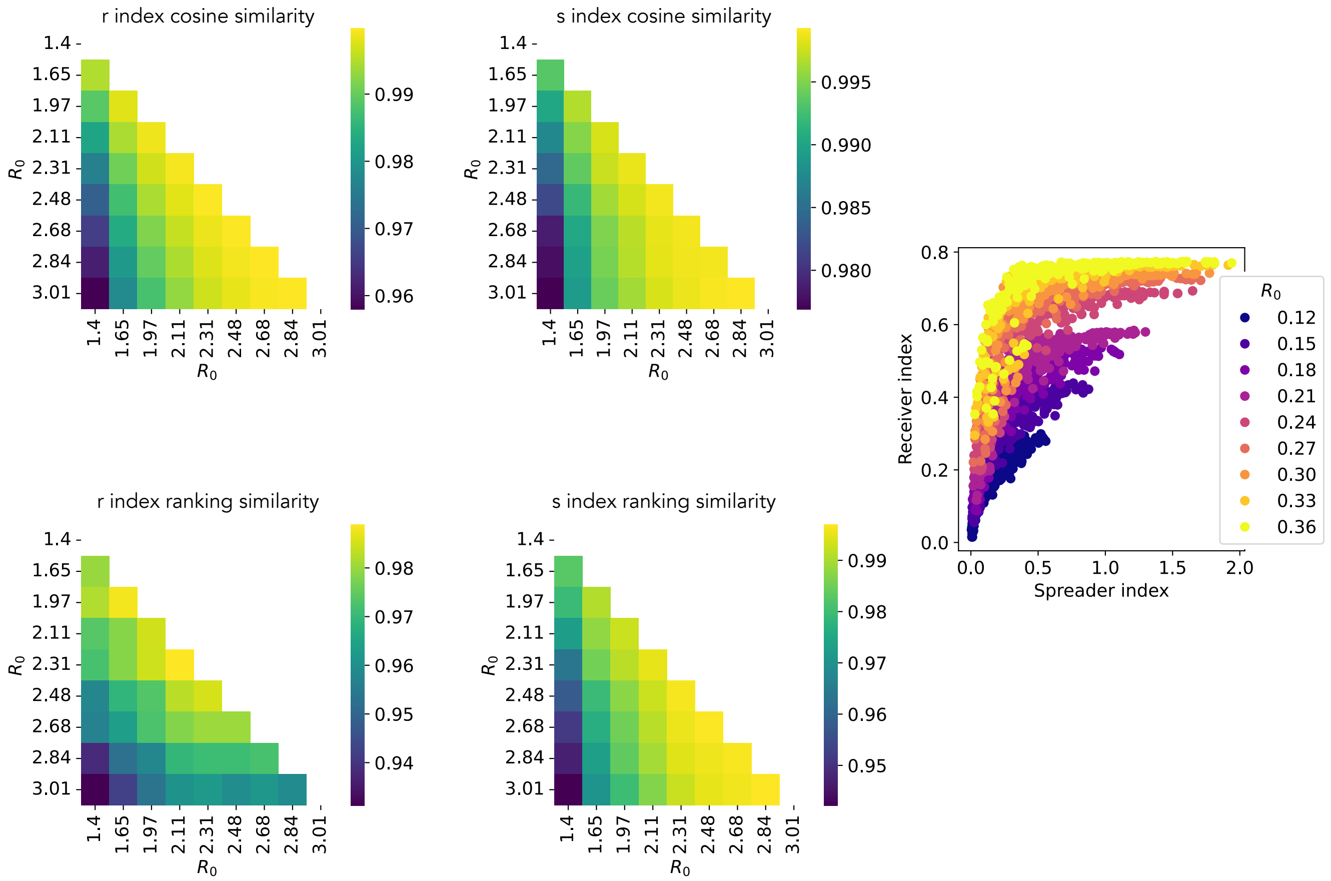

FIG. S7. Receiver and spreader indices across values of R_0 in simple contagion (SIR model) for the primary school data set.

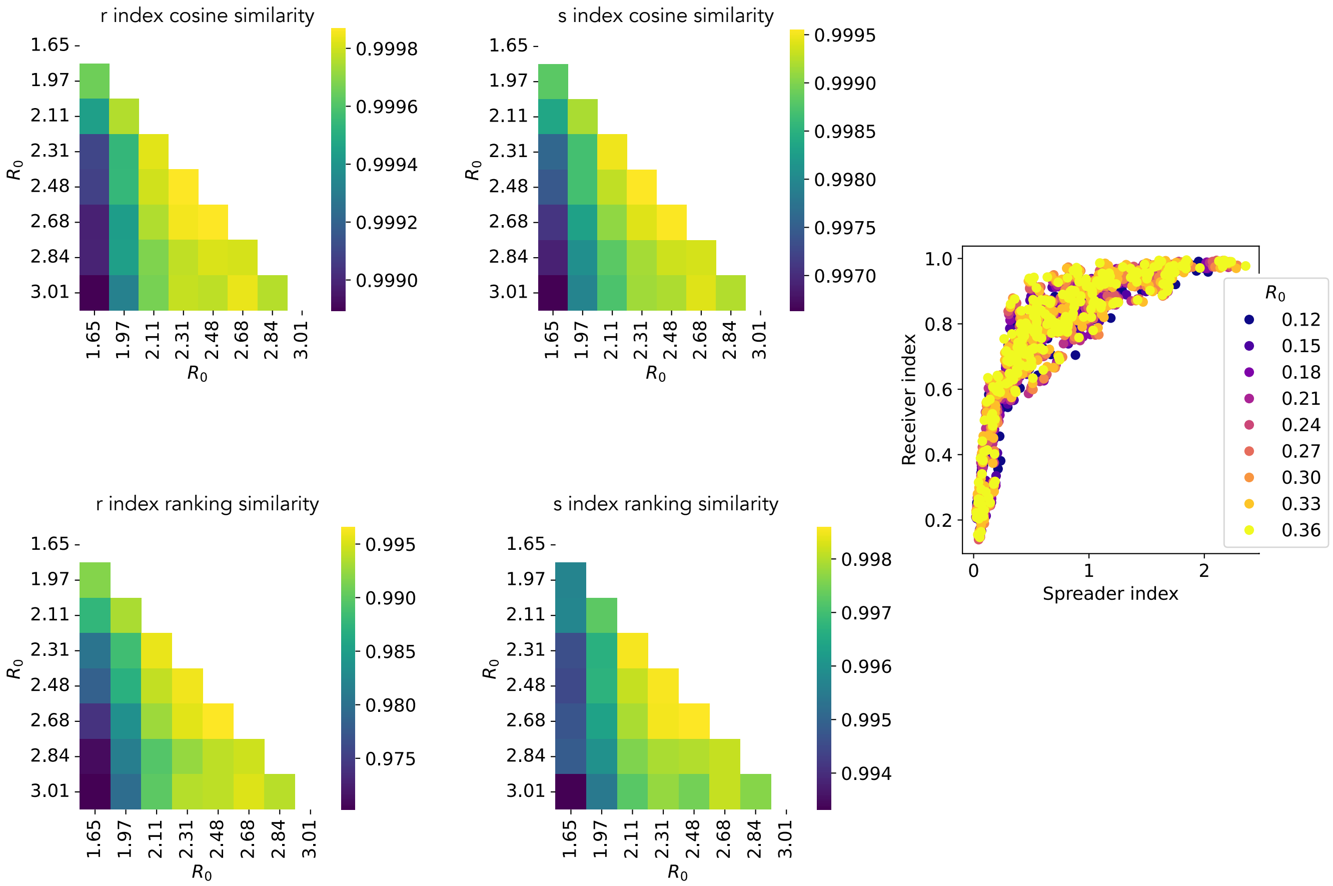

FIG. S8. Receiver and spreader indices across values of R_0 in simple contagion (SIR model) with fixed attack rate $0.75 < a < 0.85$ for the primary school data set.

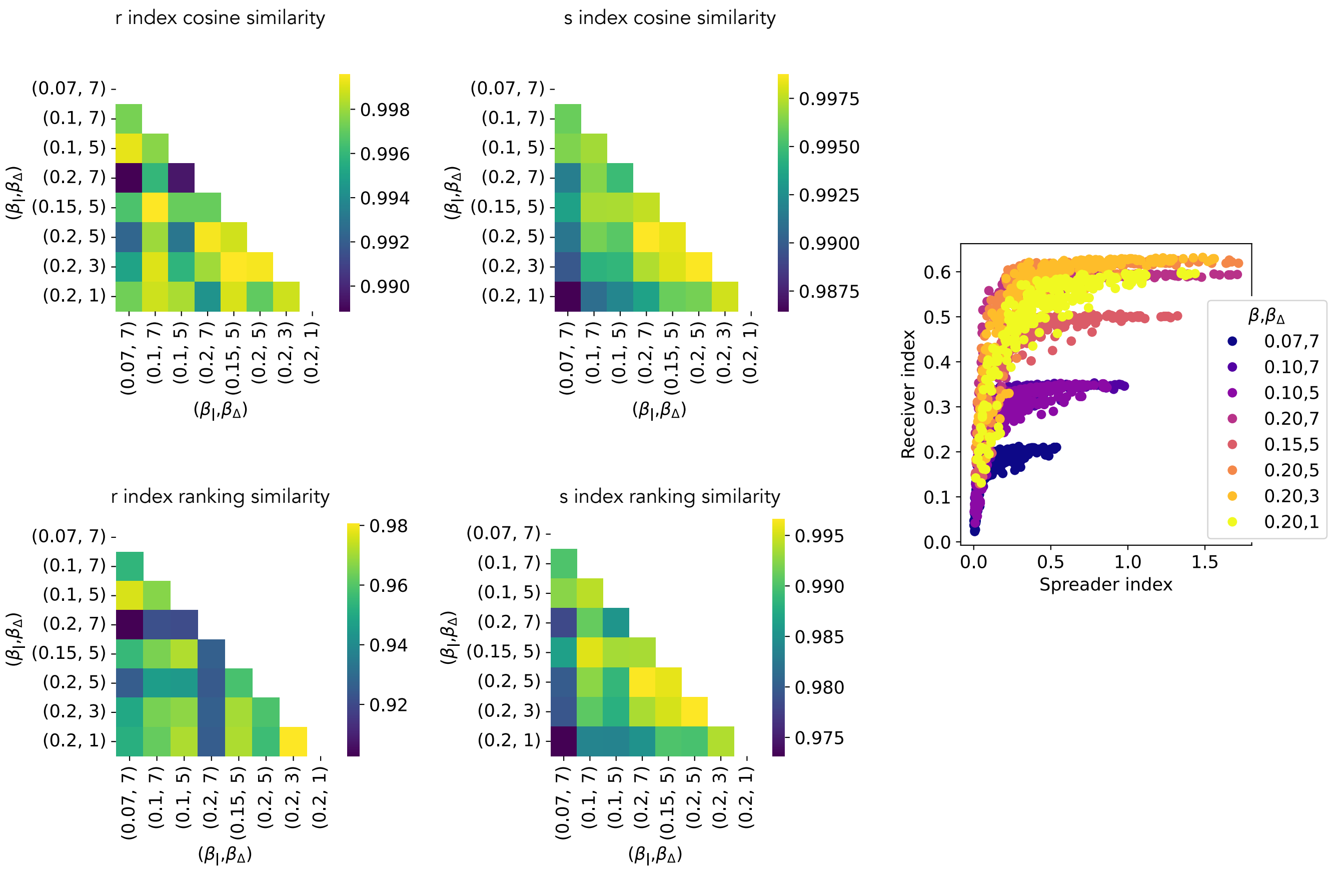

FIG. S9. Receiver and spreader indices in simplicial contagion for the primary school data set.

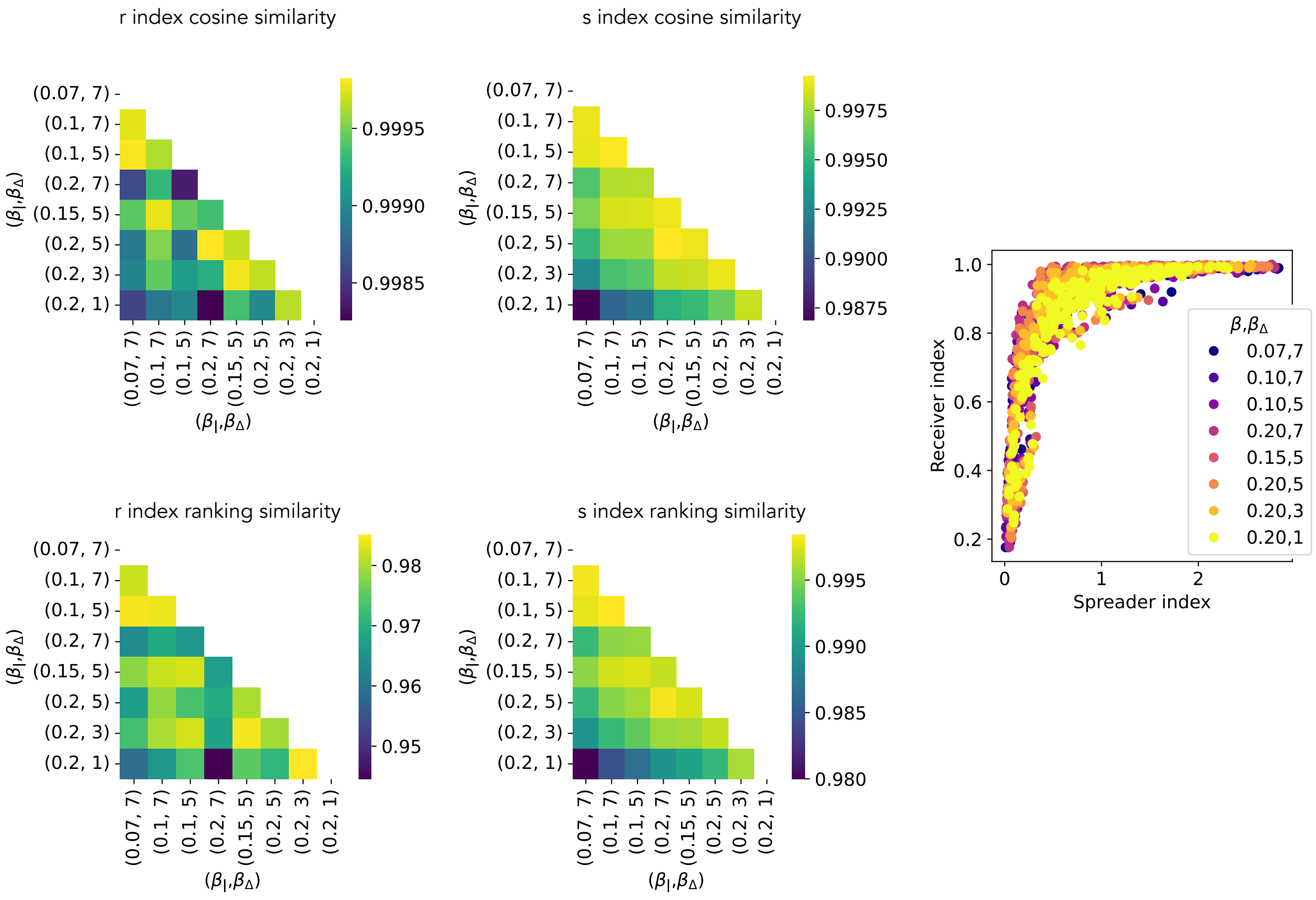

FIG. S10. Receiver and spreader indices in simplicial contagion with fixed attack rate $0.8 < a < 0.9$ for the primary school data set.

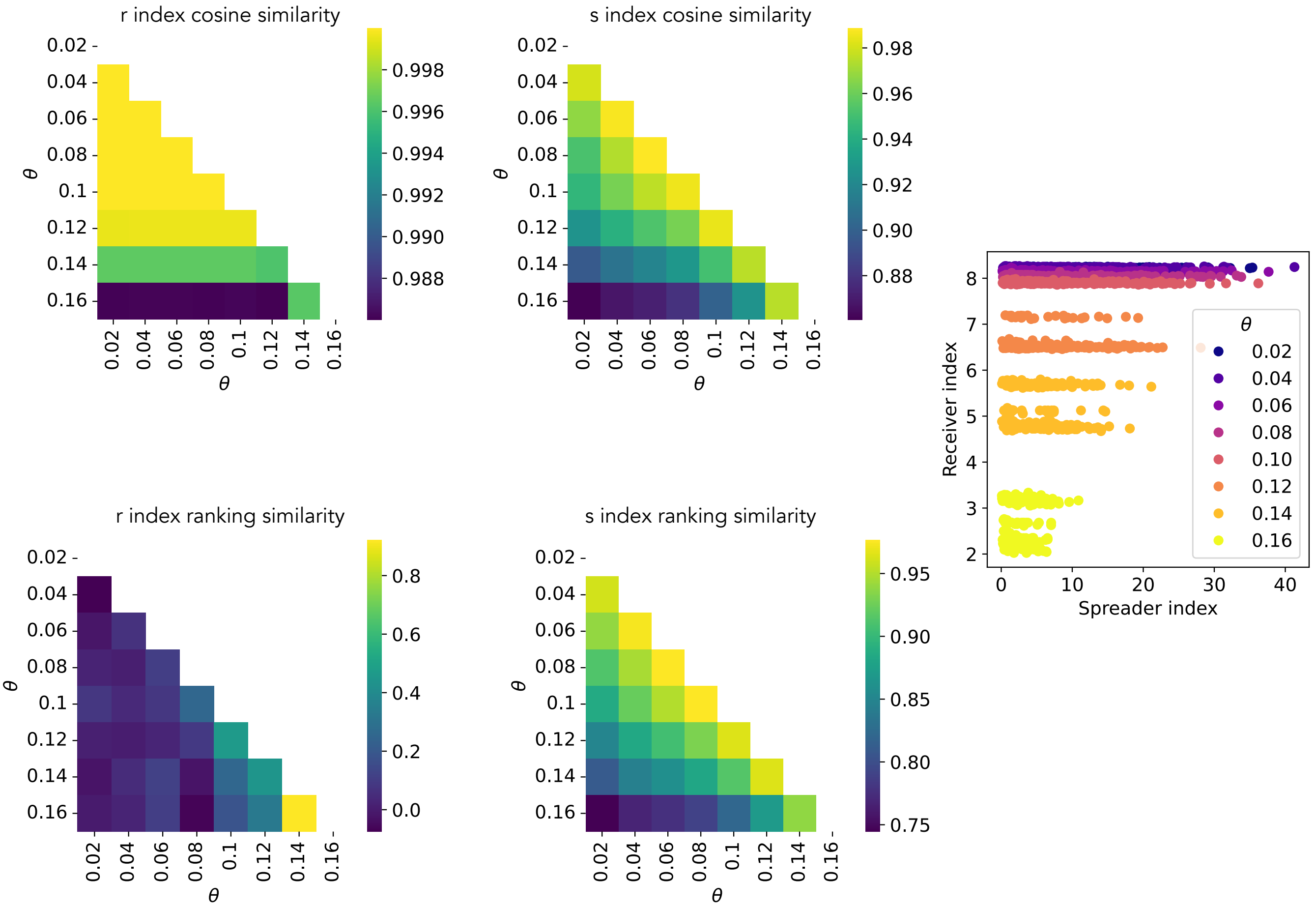

FIG. S11. Receiver and spreader indices in threshold contagion for the primary school data set. The low values of the ranking similarity for the r index are due to the fact that r spans a very narrow interval of values for each θ (see scatterplot), i.e., there is very little heterogeneity in the indices of different nodes.

G. RESULTS FOR OTHER DATA SETS

In the following figures we report the results on infection pattern similarity (for the different models of contagion and across models) for simulations performed on four additional networks of face-to-face interactions, measured in a conference [2], a hospital [3], a workplace [2] and a high school [4]. The results are in line with those of the primary school contact network presented in the main text.

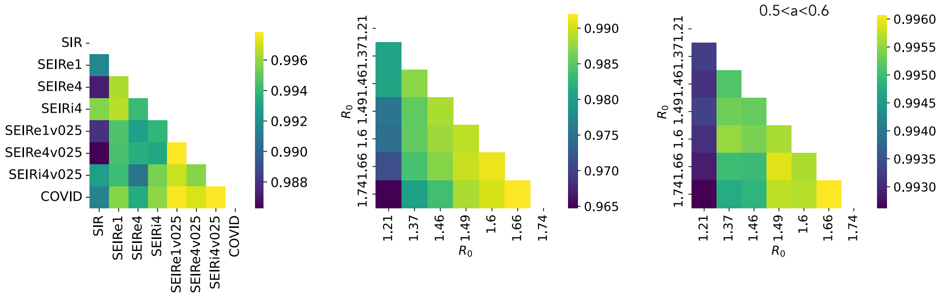

(a)Conference

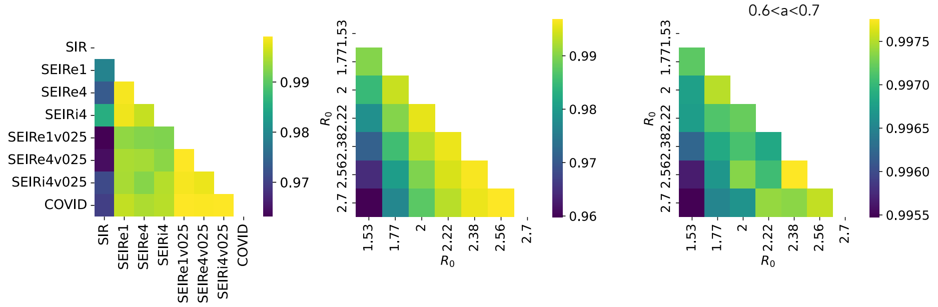

(b)Hospital

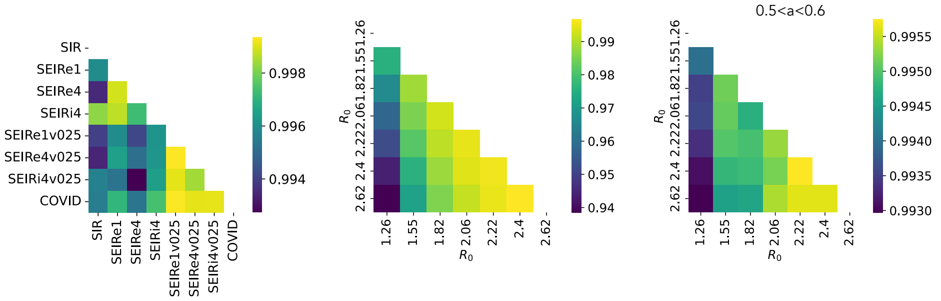

(c)Workplace

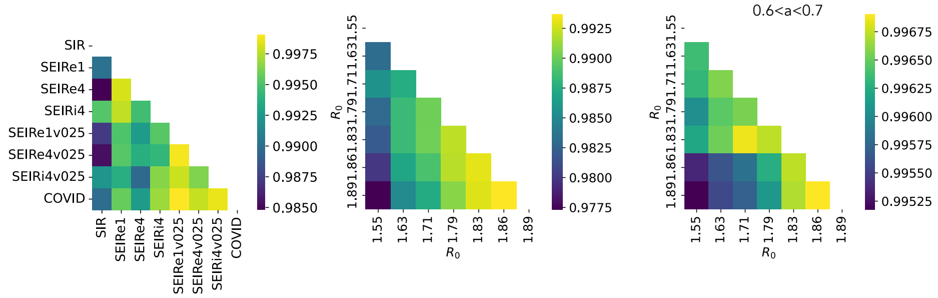

(d)High school

FIG. S12. Infection patterns in simple contagion. For each data set, the panel on the left reports the infection pattern similarity between different models of simple contagion (with $R_0 = 2.5$), the central panel the similarity between patterns obtained at different values of R_0 (with the SIR model), and the panel on the right shows the same but at fixed attack rate.

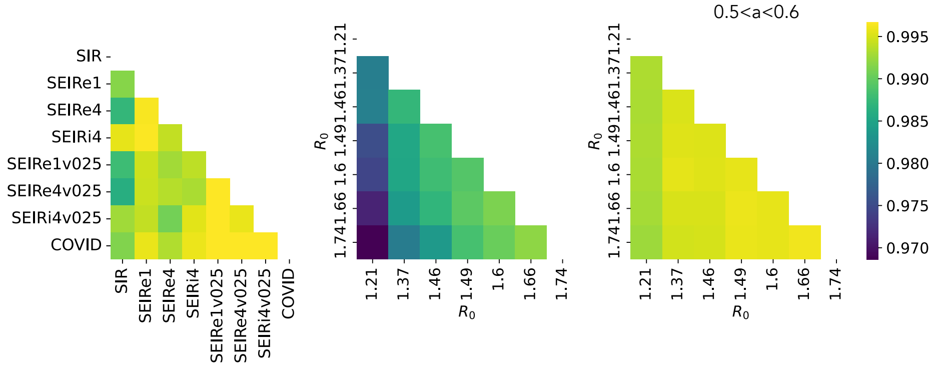

(a)Conference

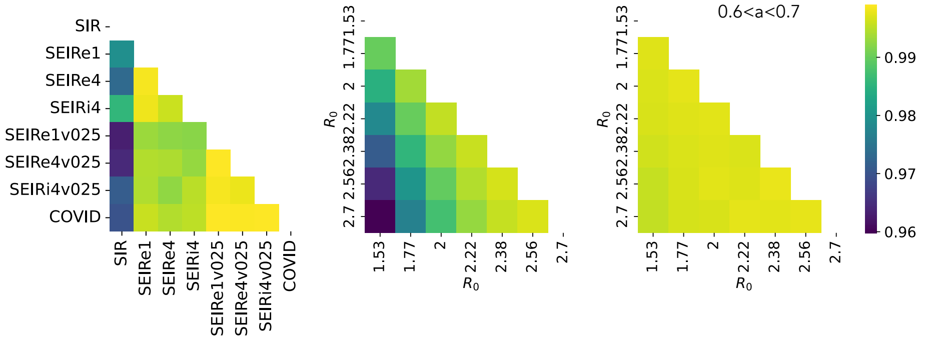

(b)Hospital

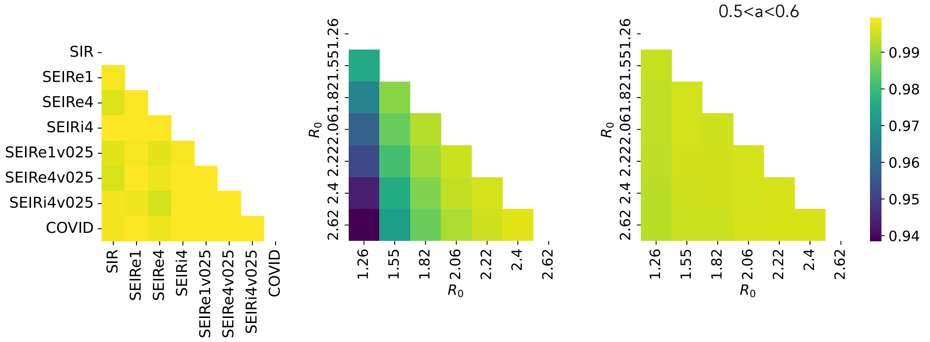

(c)Workplace

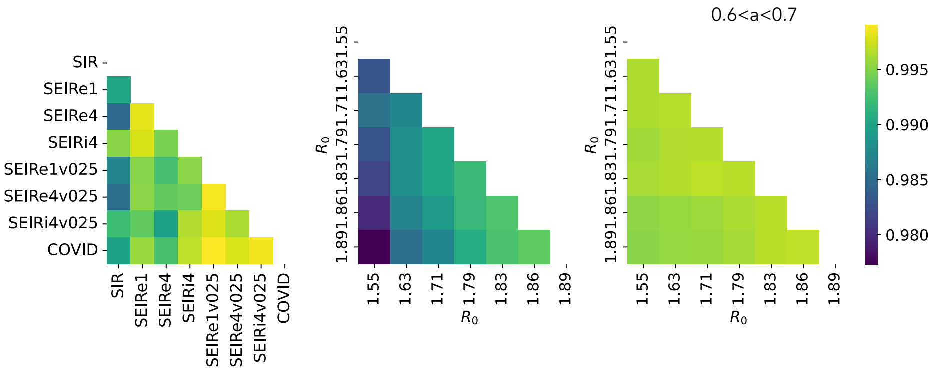

(d)High school

FIG. S13. Same as Fig. S12 with the same colorscale in all panels of a given data set.

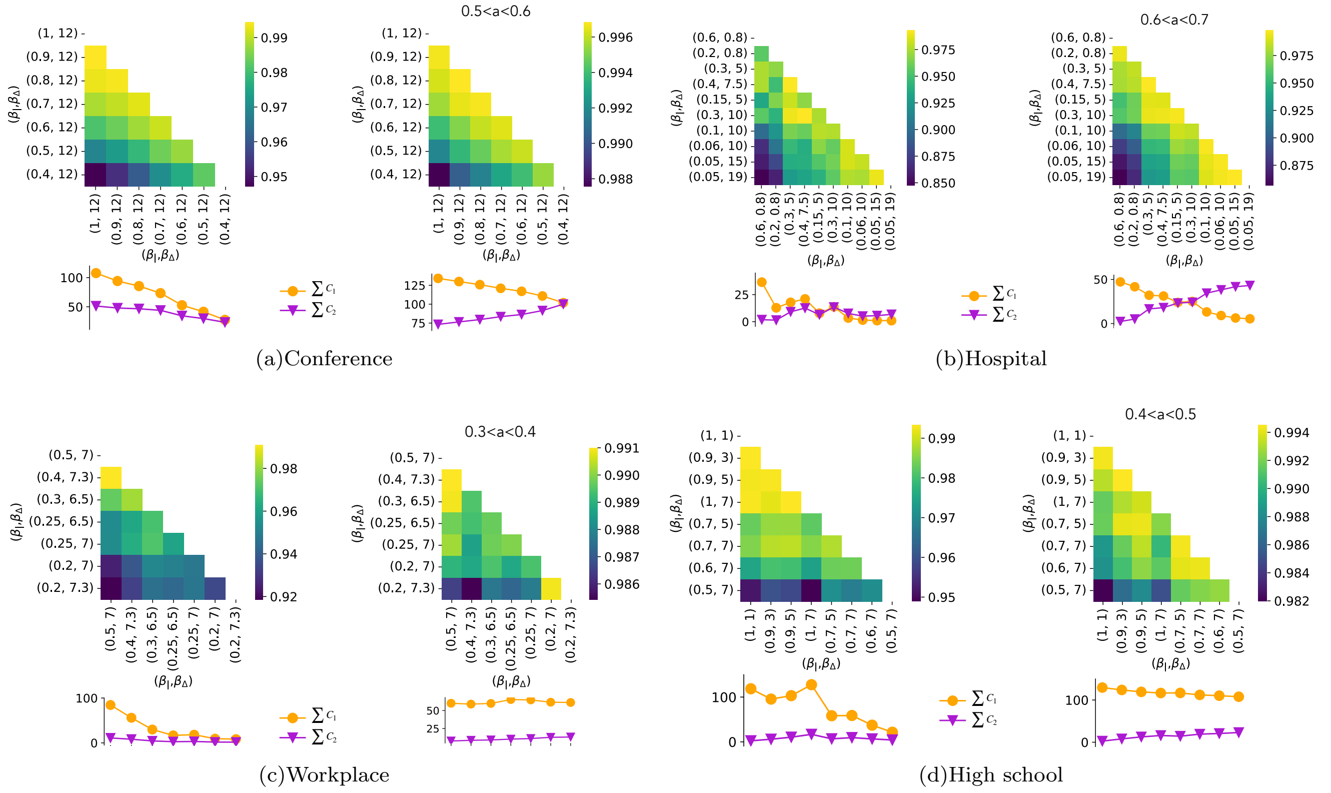

FIG. S14. Infection patterns in simplicial contagion. For each data set, the panel on the upper left reports the infection pattern similarity for different values of $\beta_|$ and β_Δ, just below the number of contagions taking place via first and second order simplices is depicted. The panels on the right show the infection pattern similarity and numbers of first and second order interactions when the attack rate is fixed.

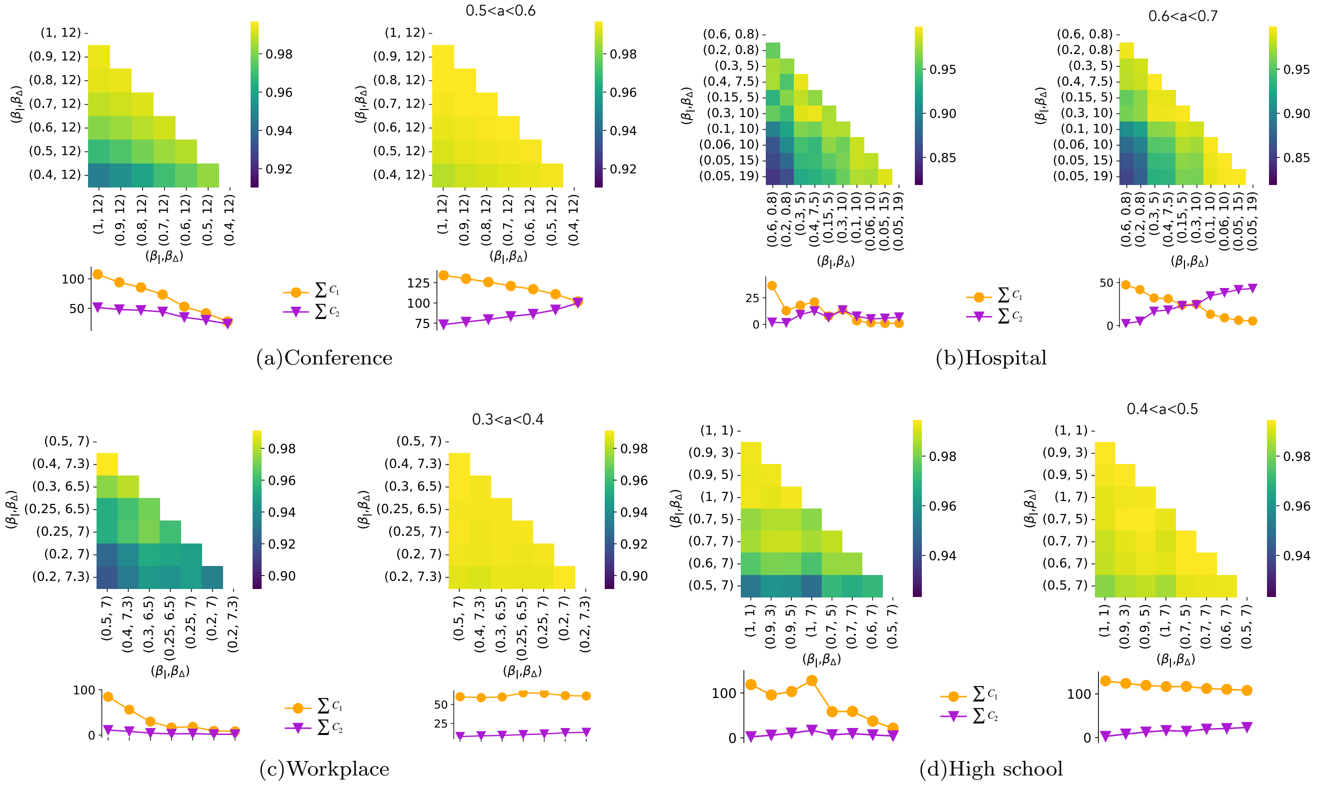

FIG. S15. Same as Fig. S14 with the same colorscale in all panels of a given data set.

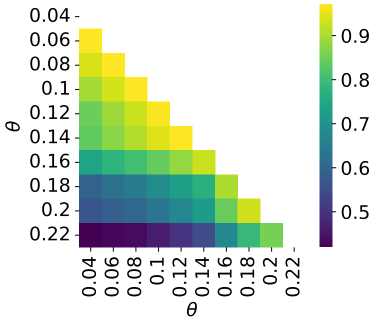

(a)Conference

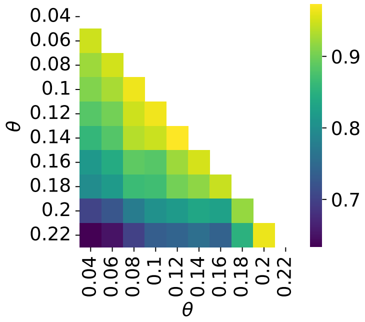

(b)Hospital

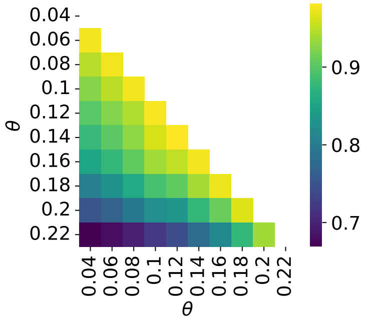

(c)Workplace

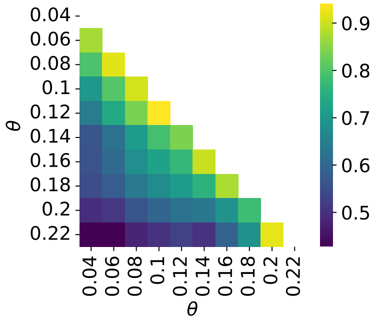

(d)High school

FIG. S16. Infection patterns in threshold contagion: similarity between the patterns obtained with different values of θ and for different data sets.

[1] Radicchi F, Castellano C. Fundamental difference between superblockers and superspreaders in networks. Phys Rev E. 2017;95:012318. doi:10.1103/PhysRevE.95.012318.

[2] Génois M, Barrat A. Can co-location be used as a proxy for face-to-face contacts? EPJ Data Science. 2018;7(1):11. doi:10.1140/epjds/s13688-018-0140-1.

[3] Vanhems P, Barrat A, Cattuto C, Pinton JF, Khanafer N, Régis C, et al. Estimating Potential Infection Transmission Routes in Hospital Wards Using Wearable Proximity Sensors. PLoS ONE. 2013;8(9):e73970. doi:10.1371/journal.pone.0073970.

[4] Mastrandrea R, Fournet J, Barrat A. Contact patterns in a high school: a comparison between data collected using wearable sensors, contact diaries and friendship surveys. PloS one. 2015;10(9):e0136497.
