# Supplementary material for: Infection patterns in simple and complex contagion processes on networks
Source: PLoS Comput Biol. 2024 Jun 10;20(6):e1012206. doi: 10.1371/journal.pcbi.1012206 (PMC11192313; doi:10.1371/journal.pcbi.1012206)
Supplement: Attachment — Submitted filename: rebuttal.pdf [file pcbi.1012206.s002.pdf]

Dear editors,

we are grateful to both reviewers for their thoughtful and constructive reviews of our manuscript. The first reviewer writes particularly positive comments and recommends publication. They make interesting suggestions that we have followed to strengthen our manuscript. In particular, their point (2) has led us to add new numerical simulations on a reshuffled version of the data, making one of our results stronger.

The second reviewer also considers our manuscript "interesting and well-written" and asks for several clarifications. We are grateful for their insightful comments that have helped us make our manuscript much clearer. We have added comments and rewritten some parts of the Methods and Supplementary Material, and added some numerical simulations to strengthen our results. We have also largely reshaped the discussion section to better highlight the concrete implications of our results.

In the following, more detailed point-by-point answers to the reviewers' concerns are reported. We hope that, with these modifications, the new version of our manuscript can be considered suitable for publication in PLOS Computational Biology.

Sincerely yours,

Diego Andrés Contreras, Giulia Cencetti, Alain Barrat

Detailed answers to the Reviewers' comments

Reviewer 1

- *Contreras, Cencetti, and Barrat have submitted a manuscript entitled "Infection patterns in simple and complex contagion processes on networks" for publication in PLOS Computational Biology. Overall, this is a great numerical simulation paper in computational epidemiology. Modulo some notes below, publication in PLOS Computational Biology is clearly warranted. When I consider the contributions of the paper, I see the following primary points:*
  (a) A clear formulation of problem, with numerical insights closely associated with (and thoroughly explored through) the model parameters
  (b) The use of empirical datasets—and, when non-trivial patterns do occur, the explanations with regards to the particular features of the datasets.
  (c) A simple-to-understand story line: Simple infection patterns are largely similar, but group contagions depend on an interplay between pairwise and higher-order parameters. The threshold contagion may be made similar to the simple one, but only when the threshold parameter is large.

  Answer: we warmly thank the Reviewer for these very positive comments on our work.

- *Notably not on the list are: (1) The abstract is on the lengthy end. It would be nice if the authors could trim it to 1/2 to 2/3 of the current size.*

  Answer: we have shortened the abstract to about 2/3 of its previous length.

- *(2) In Section II-C, and regarding (b) above, it would be nice if the authors could supplement a contrasting numerical experiment, based on the same scaffold (i.e., same edges) of an empirical social network, but artificially redesign the weights in some way, such that the higher-order interactions and the pairwise ones do not necessarily overlap, and re-run the experiments at the end. This is to test whether the explanation—that both simple and higher-order mechanisms tend to use the same infection routes, when the weights are correlated—is probable or not.*

  (3) Adding to (2), this kind of dataset could be a network where participants spend time with families and friends, but also a significant time with colleagues. With colleagues (who are not friends or family members), the W_{ijk} is large, but the pairwise W_{ij}, W_{jk}, W_{ik} are small. I am not sure if sociopatterns contain this type of dataset.

  Answer: the SocioPatterns data sets concern each only one specific context, and therefore do unfortunately not contain data in which contacts of the same individual in different contexts such as friends and work are registered.

  The point raised by the Reviewer is however of great interest indeed, and we have designed the following procedure to test our explanation. First,

we have generated a synthetic hypergraph obtained from the real one by randomizing the link weights, while keeping fixed the weights of the hyperedges of larger size. In this way, the structure of the hypergraph is fixed (so links and triangles still overlap), but the correlation between the weights on the links and the weights on the triangles are removed. Then, we have simulated the contagion processes and reported the results for the infection patterns in fig. S4 of the supplementary material. Similarity values are notably lower than with the original weights. This result confirms that the high similarity observed in the real hypergraphs is largely due to correlations. We have added a section in the supplementary material to show these results, and have added a reference to it in the main text.

- *(4) In the Lines between 354-362 (in Section II-C), is there a reason that the authors choose 1/2 and 1/2 for the contributing C_{ij} weights. Why not choose numbers (which sums to 1) with proportion to W_{ij} and Wlj?*

  Answer: there is a distinction between the roles of i and l in the contagion of j if and only if the contagion occurs through a binary event. If the contagion occurs due to a triadic interaction ijl with both i and l infected, i and l play exactly the same role in this event, and therefore the "responsability" of the contagion is equally divided between both. We have added a comment to make this clearer.

- *(5) In the last paragraph of Section II-D, the sentences are not very comprehensible to me. For example, what does the *it* in Line 475 refer to? (I confess that I jumped to reading this paragraph, and noticed that I did not understand its meaning.)*

  Answer: We have rephrased the paragraph to make it clearer.

- *(6) Please note that all the points (1)-(5) are minor.*

  Answer: we are grateful to the Reviewer as these points have helped us make our results stronger and clearer.

Reviewer 2

- *To the authors of the manuscript titled "Infection patterns in simple and complex contagion processes on networks",*
  In this manuscript, the authors aim to understand the differences in infection patterns for given network structures using different contagion models. The manuscript is interesting and well-written, however, there are important points that need clarification or elaboration:

  Answer: we thank the Reviewer for this careful reading of the manuscript and this positive appreciation of our work. We are grateful for the detailed comments, to which we answer in detail below, and that have helped us make our manuscript clearer and better emphasize the relevance of our results.

- *1. The Discussion section of the study requires significant elaboration on the implications of the results and findings. The authors are expected to address how other researchers, decision makers, and/or policy makers can use and derive benefits from these findings. Furthermore, the authors can consider mentioning the broader implications for the infectious diseases research community.*
  2. The contribution of the findings of this study to the existing knowledge base needs elaboration.

  Answer: We have largely rewritten the discussion section to make clearer how our results contribute to the state of the art and can impact other research. In particular, we have separated in the discussion a first global and factual summary of our results and then a discussion about their implications on the way numerical simulations of spreading processes should be envisioned.

  Indeed, our results imply that crucial information regarding the infection patterns of a disease can be gained from simulations based on very simple models, and without an important dependence on parameters. This is very important as, when a new disease emerges, it is initially difficult to estimate its parameters and sometimes even the types of compartments that should be taken into account in its modeling.

  This also means that it is possible to attribute a classification of the nodes depending on their risk to be infected and on their spreading power, which once again almost does not depend on the disease being considered, and which can thus be determined using simplified models.

  Finally, even if complex and simple contagion processes have different mechanisms, their patterns remain partly similar, which implies that a partial information can be gained from simple models even on the way complex contagion processes unfold, although the uncertainty is higher. Moreover, these differences highlight the need for data collection efforts on higher order interactions, to correctly inform the complex contagion models.

  We have also slightly reshaped the introduction part to highlight the bigger issue of the applicability of information gained from simulations of simple models to more realistic cases.

- *3. The Methods section, in general, is expected to provide more information for understanding the findings and reproducing the results presented: 3.1. The set of equations, pseudocodes or visualizations of the simulation models would enhance comprehension of the model structures. These models may be intuitive to many researchers but require elaboration given the broader audience of PLOS CB.*

  Answer: we have added a figure (figure 1) sketching the contagion mechanisms of each model. Moreover, our code is also now made available on a github repository.

- *3.2. The selection of the Model Parameters in Table 1 needs explanation.*

  Answer: Given the network is static, we first note that the important parameter is R_0, and that we can rescale all parameters by an arbitrary factor, which would change the overall timescale of the process but not its dynamics.

  For the SIR and SEIR models, we have considered values that are all of order 1 for simplicity. Moreover, we have considered several versions with different values of the average durations of the exposed and infected states, and we have considered a factor 4 between the values considered so that they differ significantly but do not change order of magnitude (which would be unrealistic).

  For the non-Markovian cases, we have also there considered values of the factor η of 0.25 in order to have a clearly different distribution of the durations of the states with respect to the Markovian case, without going to extreme, unrealistic cases.

  For the COVID model finally, we have used parameters taken from previous literature, as referenced in the text.

  We have added a justification of our choices in the Methods.

- *3.3 How the parameter beta is tuned to obtain a desired specific value for the basic reproduction number R0 needs elaboration. 3.4. The calibration process under the heading "B. Reproductive number and calibration" requires clarification.*

  Answer: We now provide details on the procedure used to calibrate the parameter beta in the subsection "Reproductive number and calibration" of Methods section.

- *3.5. Among the 5 datasets (primary school, workplace, hospital, high school and conference), the rationale behind selecting the primary school dataset for the main body of the manuscript needs elaboration.*

  Answer: this data set has a direct relevance to public health questions, given the importance of schools in the context of infectious diseases, and has indeed been used in a number of works published in epidemiology/public health journals as a support for numerical simulations aiming e.g. at the evaluation of containment strategies in school settings. Its structural and temporal properties have also been widely studied, and it has a number of interesting features such as clear communities (the classes) in the aggregated network, which however fluctuate temporarily since students of different classes mingle during the breaks. Moreover, it features an important number of simultaneous group interactions, which makes it an interesting data set to study contagion processes involving both pairwise interactions and larger groups. We have added some comments in the text to justify our choice.

- *3.6. The calculations for the weights of the links between nodes need elaboration. What are the assumptions behind these calculations? Could you provide the steps required to create the same weighted graphs using the dataset references you provided?*

  Answer: We have updated the Methods section, changing the section "Weighted hypergraphs" into a section "From the data to weighted graphs and hypergraphs" in which we explain in details the procedure to go from the publicly available data sets to the weighted graphs and hypergraphs we use.

- *3.7. As far as I understand, the authors use weighted graphs for "simple models", while they use weighted hypergraphs for "simplicial models" and "threshold models". If this is the case, it is expected to be explicitly stated in Methods.*

  Answer: The weighted graphs are used both for simple models and threshold models, in which the contagion events use only binary interactions. The weighted hypergraphs are used for the simplicial models, where contagion events occur either along links or along hyperedges of size 3. We now state this explicitly in the section "From the data to weighted graphs and hypergraphs".

- *3.8. Again, as far as I understand, weighted graphs are formed by using the average daily time between contacts, while weighted hypergraphs are formed by using the number of times that the connection has appeared in the data. The authors are expected to explain the reasoning behind this difference, and any observations they find resulting from this difference.*

  Answer: as the data sets have a constant temporal resolution, the daily duration of the interaction between two nodes is simply the number of times that the connection appears during that day, multiplied by the temporal resolution (20 seconds). There is thus only a factor between the two types of weights. As the networks considered are static, the resulting dynamics are thus completely equivalent, up to a global temporal rescaling. To make things simpler, we now use the same convention in all cases, and have modified the text accordingly: the weight of a link is equal to the number of times the connection appears in the data, normalized by the maximum observed weight.

- *3.9. The general idea/formula for cosine similarity is required.*

  Answer: We added a section in Methods titled "Cosine similarity".

- *3.10. Last but not least, the platform on which these simulations and analyses were conducted is expected to be stated. The simulation models should be able to be run/repeatable by other researchers.*

  Answer: all the codes were written in Python, and are now available.

- *4. Page 3, lines 275-277: “This suggests that the infection pattern of a spreading model mostly depends on its average final attack rate.” I believe that this statement may be overly strong, since this is an observation for a given range of attack rates. For instance, what percentage of the simulations fall into the range* $0.75 < a < 0.85$*? Do the authors observe the same phenomena for other ranges of attack rates as well? (This may bring us to the following point). I believe the generalizability of this statement needs to be reconsidered.*

  Answer: for each value of R_0, the attack rate varies depending on the specific stochastic simulation. We obtain thus at each R_0 a distribution of attack rates, which we now show in the Supplementary Material. To compare infection patterns of different R_0 for the same range of attack rates, we thus need that these attack rates are effectively obtained for the R_0 values considered. This is why we had shown only the range $0.75 < a < 0.85$, which is, as seen from the distributions in the SM, the most accessible for the R_0 values we had considered. In order to check what happens for other attack rates, we have now performed additional simulations using other values of R_0, and we show the additional results in the new version of the figure (now figure 3). We obtain, also in the new ranges of attack rates, that the similarities between infection patterns at different R_0 become extremely high when the attack rate is fixed, generalizing the result.

  We have updated the main text to comment the new version of the figure and the new results. We have also added a section in the SM titled “Distributions of final attack rates in the SIR simple contagion model”, in which we show the distribution of attack rates for the various values of R_0 considered.

- *5. Page 4, lines 307 – 310: ”In other words, at each time step the infection pattern (which describes the contagion probability of each connection until that time) is almost completely determined by the attack rate reached at that specific time.” I, unfortunately, could not fully comprehend that conclusion. Some clarification on the construction of Figure 3 may help other readers to understand that statement. Do results in Figure 3 correspond to the simulation of 1000 runs, or less, or just 1 example run for each panel and each R0? How were the distributions of attack rates at the bottom of Figure 3 formed? Were the distributions formed using multiple runs? In general, the chain of thought from the observations in Figure 3 to the conclusion stated in lines 307-310 needs further clarification.*

  Answer: For each R_0, we always consider 1000 simulations to build the infection patterns, and all the attack rate distributions are also built using 1000 simulations. For $R_0 = 4$, we follow the distribution of attack rates over time, by considering at each time t the attack rates reached at that time and building the corresponding distribution. The middle panel shows the time evolution of the non-zero mode of these attack rate distributions. We also build the time-dependent infection patterns $C_{ref}(t)$ by averaging

over all contagion events that have occurred until t.

For the other values of R_0, we instead let 1000 simulations run until the end for each R_0 and compute the resulting infection patterns C_{R_0} and final attack rate distributions. The top panel of the figure shows the similarity between $C_{ref}(t)$ and C_{R_0} versus time for each R_0, and the middle and bottom panels show that the maximum is obtained when the attack rates of the reference simulation is close to the final attack rate of the testing R_0 values.

We have explained the procedure in more details, largely rephrasing the part describing the figure (now figure 4). The important conclusion is that one can use a single large value of R_0 to gain access to infection patterns at lower R_0.

- *6. A visualization demonstrating how simplicial contagion models look would be helpful for the readers.*

  Answer: we have added a figure (new figure 1) as explanatory sketches for all contagion models considered.
