# Supplementary material for: Infection patterns in simple and complex contagion processes on networks
Source: PLoS Comput Biol. 2024 Jun 10;20(6):e1012206. doi: 10.1371/journal.pcbi.1012206 (PMC11192313; doi:10.1371/journal.pcbi.1012206)
Supplement: Attachment — Submitted filename: rebuttal.pdf [file pcbi.1012206.s003.pdf]

Dear editors,

thank you for obtaining the reports on the revised version of our manuscript titled "Infection patterns in simple and complex contagion processes on networks". We are very glad that both reviewers have appreciated our efforts to change the manuscript in response to their comments, and that they feel that the manuscript has improved and is now more accessible.

The second Reviewer had some additional comments, which we have now addressed. First, we would like to apologize for the fact that the code was not easily usable. We have corrected this issue and enriched the README file as suggested by the Reviewer. Second, we have enriched the Discussion section to relate each result to the previous literature in a clearer way. We hope that, with these modifications, this new version of our manuscript can be considered suitable for publication in PLOS Computational Biology.

Sincerely yours,

Diego Andrés Contreras, Giulia Cencetti, Alain Barrat

Detailed answers to the Reviewers' comments

Reviewer 2

- I thank the authors for addressing the comments so carefully and thoroughly. I believe the revision of the manuscript has significantly improved its comprehensibility, making it more accessible to a wider audience. I have some additional comments about the manuscript:

  Answer: we are grateful for this positive appreciation of our efforts to revise and improve the manuscript, following the suggestions of both Reviewers.

- Even though the authors showed significant effort in editing the Discussion section, I think the comment saying "The contribution of the findings of this study to the existing knowledge base needs elaboration." has not been addressed yet. The authors are suggested to elaborate on the interplay between their findings and the available literature and summarize how their findings align with or oppose the current knowledge. In the current version of the Discussion section, the relationship between the manuscript's implications and the existing studies has not been presented, which makes it harder for the readers to comprehend the concrete contribution of the work done by the authors.

  Answer: in the previous version of the manuscript, we had added a number of references and described the state of the art in the introduction, and added comments about the implications of our results for decision making and modeling purposes. The Reviewer is however right that we had not discussed directly the link between our results and the relevant literature. We have therefore rewritten the paragraph of the discussion summarizing our results, in order to relate each result to the current body of knowledge. It is particularly interesting to note that some of our results allow in fact to put on firmer ground a body of literature concerning the relation between topological centrality measures and spreading power or location of sentinel nodes, which is most often discussed using arbitrary spreading models and parameters. Moreover, for what regards complex contagion, we note that to the best of our knowledge we are the first to investigate the robustness of the spreading patterns.

- The code for the models are available on Github but the models cannot be run (at least by me), so the results on the manuscript are not reproducible. While trying to run the models, I got errors because of missing files or functions. The authors are suggested to enrich the ReadMe fie on the repository with a summary of instructions on how to reproduce the findings presented in the manuscript.

  Answer: we apologize for the fact that the code was not easily usable. We have corrected a couple of errors and checked carefully that the code can run once downloaded. More importantly, we have rewritten the README

file to explain how to reproduce step by step several of the manuscript results and figures.

- Minor comment: In Section S7, the references for the datasets are broken and shown with [?].

  Answer: we have corrected this mistake.
